# Fully Digital versus Conventional Workflows for Fabricating Posterior Three-Unit Implant-Supported Reconstructions: A Prospective Crossover Clinical Trial

**DOI:** 10.3390/ijerph191811456

**Published:** 2022-09-12

**Authors:** Ali Mahmoud Hashemi, Hamid Mahmoud Hashemi, Hakimeh Siadat, Ahmadreza Shamshiri, Kelvin Ian Afrashtehfar, Marzieh Alikhasi

**Affiliations:** 1 Dental Implant Research Center, Dentistry Research Institute, Tehran University of Medical Sciences, Tehran 1417614411, Iran; 2 Department of Oral and Maxillofacial Surgery, School of Dentistry, Tehran University of Medical Sciences, Tehran 1411713135, Iran; 3 Department of Prosthodontics, Tehran University of Medical Sciences, Tehran 1411713135, Iran; 4 Department of Epidemiology and Biostatistics, School of Public Health and Institute of Public Health Research, Tehran University of Medical Sciences, Tehran 1417614411, Iran; 5 Department of Reconstructive Dentistry and Gerodontology, School of Dental Medicine, Faculty of Medicine, University of Bern, 3010 Berne, Switzerland; 6 Division of Restorative Dental Sciences, Clinical Sciences Department, College of Dentistry, Ajman University, Ajman City P.O. Box 346, United Arab Emirates

**Keywords:** computer-aided design, implant-supported dental prosthesis, dental impression technique, patient satisfaction, CAD-CAM

## Abstract

This study assessed the clinical variables influencing the success of three-unit implant-supported fixed dental prostheses (ISFDPs) fabricated using either fully digital or conventional workflows. The clinical trial evaluated 10 patients requiring three-unit ISFDPs in the posterior mandible. Maxillomandibular relation records, and digital and conventional impressions were obtained from each patient using an intraoral scanner (IoS) and polyvinylsiloxane (PVS), and the frameworks were fabricated using zirconia and cobalt–chromium, respectively. A 2 µm accuracy scanner scanned the conventional master casts and standard reference models. The stereolithography (STL) files of the digital and conventional impressions were superimposed on the standard model file, and the accuracy was calculated with the best-fit algorithm. The framework adaptation and passivity were assessed using the one-screw and screw resistance tests. The time required for occlusal adjustment of both types of reconstructions, including the duration of the whole treatment, was recorded. The aesthetic appearance of ISFDPs was rated by each patient and clinician using a self-administered visual analogue scale questionnaire and the FDI World Dental Federation aesthetic parameters, respectively. The sample size was based on the power calculation, and alpha was set at 0.05 for the statistical analyses. The impression accuracy, framework adaptation and passivity, and reconstructions aesthetics did not significantly differ between the digital and conventional approaches. The total fabrication time was significantly shorter using the digital workflow. Within the limitations of this clinical study, the fully digital workflow can be used for the fabrication of ISFDPs with a clinical outcome comparable to that of the conventional workflow.

## 1. Introduction

Digital technology has revolutionized the personal and professional lives of most people worldwide [1]. For years, many strategies have been proposed for advancements in implant dentistry [2]. The digital technology enabled surgical guides to precisely determine the implant site in dental implant surgery [3,4]. Computer-aided design–computer-aided manufacturing (CAD–CAM) technology is a well-established technology for manufacturing implant-supported fixed dental reconstructions [5]. Accurate intraoral scanners (IoS) with their respective software programs enable precise fabrication of restorations without requiring impression materials or complex laboratory procedures [6,7].

There is still a dilemma between the use of the conventional or digital workflow for the fabrication of implant-supported reconstructions [8,9,10]. Thus, selecting the digital or conventional workflow for implant-supported fixed dental prostheses (ISFDPs) fabrication can be challenging for many dental clinicians. In addition, these decisions require attention to biomedical, anatomical, aesthetic, and financial aspects of treatment [11]. Moreover, some clinicians prefer a combined analogue with digital techniques [12]. The conventional workflow for manufacturing ISFDPs is considered to present shortcomings such as a long time from start to end, high financial investment, impression materials’ distortion, and patient discomfort. In contrast, the digital workflow has minimized human manipulation errors in the entire process [13,14]. Additionally, it does not require the entire impression to be repeated in the case of a defect [15]. Moreover, digital technologies enable the three-dimensional (3D) observation of the fabrication process as well as same-day delivery of the final prostheses. All these parameters further add to the great interest in digital approaches [16,17]. 

Several in vitro studies are available regarding digital systems [18,19,20]. The scientific validation and evidence for the clinical and technical feasibility are crucial to understanding the impact of the actual digitalization trend on modifying current conventional protocols in fixed prosthodontics [11]. However, considering the differences between the in vivo and in vitro settings, clinical studies are still required in this respect. 

Since prospective clinical studies comparing digital and conventional techniques are challenging to conduct, clinical studies on this topic are limited [21,22]. Additionally, most of these studies have been conducted on natural teeth, and clinical studies on implant restorations are often limited to single-unit restorations [23,24]. Thus, this study aimed to compare the clinical outcome of fully digital and conventional workflows for the fabrication of three-unit implant-supported restorations with respect to impression accuracy, framework passivity and fitness, the aesthetics and occlusion of restorations, and the required time. The null hypothesis was that no significant difference would be found between the two workflows regarding the abovementioned parameters.

## 2. Materials and Methods

### 2.1. Ethical Approval and Trial Registration

This prospective clinical controlled trial was conducted at a university setting from October 2019 to February 2021 and followed the principles of the Declaration of Helsinki relating to biomedical research involving human subjects. The local ethics committee approved the study (IR.TUMS.VCR.REC 1398.673), which was registered (Iranian Registry of Clinical Trials: IRCT20191009045041N1). This study consisted of 10 patients requiring three-unit ISFDPs in the posterior mandible. The criteria for reporting the prospective clinical controlled study were derived from the Consolidated Standards of Reporting Trials (CONSORT) guidelines [25,26,27]. 

### 2.2. Eligibility Criteria for Participants

#### 2.2.1. Inclusion Criteria

Patients who received two regular diameter bone-level implants (ϴ4/4.5 mm, ≥8 mm of length; Dentium Co., Seoul, Korea) in posterior mandibular edentulous areas. 

Completed their recovery period. 

Able and willing to follow study instructions.

Required at least one three-unit ISFDP (fixed-pontic-fixed). 

Signed the informed consent form.

The dental implants platform had <5 mm depth relative to the gingival level. 

Inter-implant angle <10 degrees.

Inter-arch space <15 mm. 

Presence of occlusal stops (natural teeth or any type of fixed prostheses). 

#### 2.2.2. Exclusion

Patients with unstable systemic conditions.

Pregnant or lactating women.

Presence of acute dentoalveolar infections.

Untreated periodontitis.

Need for bone grafting

### 2.3. Trial Groups

A conventional and a digital impression were obtained from each patient. To standardize the level of patient cooperation and prevent patient fatigue, the sequence of impressions was randomised by the envelope technique. The antagonist casts were obtained using alginate impression material (Alginoplast, Heraeus Kulzer, Hanau, Germany) and IoS for the conventional and digital techniques, respectively. Conventional and digital static maxillomandibular records were also obtained. 

### 2.4. Experimental Group

For the digital technique, the scan body (Short length OST-GS, Arum Dentistry Co, Daejeon, Korea) was tightened (10 Ncm), and a digital impression (or intraoral scan) of the entire arch was performed with an IoS (3Shape TRIOS 3; 3Shape, København, Denmark). 

### 2.5. Control Group

The group scheduled for conventional workflow had two square-shaped impression copings (Dentium Co., Seoul, Korea) inserted on the implants for an open-tray impression technique using one-step putty-light body addition silicone (Panasil, Kettenbach GmbH & Co., Eschenburg, Germany) in a resin custom tray. After the impression, the implant analogues were secured to the impression copings before pouring gingival replica (Gi-mask, Coltene Whaledent, Altstätten, Switzerland) and type IV stone (Herostonel Vigodent Inc., Rio de Janeiro, Brazil). The cast was removed from the impression after 120 min. The working casts were scanned by an industrial scanner (Atos Core 5 Mp 80 mm; Rev. 02; GOM, Braunschweig, Germany) with 2 µm accuracy. 

### 2.6. Reference Model

For making a standard reference model for each patient, square-shaped impression copings were screwed into the implant analogues on the working cast, fabricated for conventional impression making. They were splinted with an acrylic resin (Duralay; Reliance Dental Manufacturing, Alsip, IL, USA). The splinted bar of impression copings was sectioned, creating an inter-coping gap of 0.3 to 0.5 mm, and reattached after 10 min to prevent acrylic shrinkage. In the clinic, the splint was fully seated on the implants, followed by being sectioned and reattached. Two prosthodontists (M.A., A.M.) verified that the splint jig impression copings were completely seated intraorally and radiographically. Elastomeric glue was applied to the inner surface of the custom resin open tray for taking mono-phase impressions using regular body addition silicone (Panasil, Kettenbach GmbH & Co., Eschenburg, Germany). After pouring the impression, a resin verification jig was fabricated on the master cast, and the jig was sectioned and reattached after 10 min. The jig was then transferred into the oral cavity, sectioned, and intraorally reattached. In case of discrepancy, the cast was altered and served as the reference model for the next steps [28]. The standard reference models were also scanned by an industrial scanner (Atos Core 5 Mp 80 mm; Rev. 02; GOM GmbH, Braunschweig, Germany). 

### 2.7. Measurement

**Figure 1:**
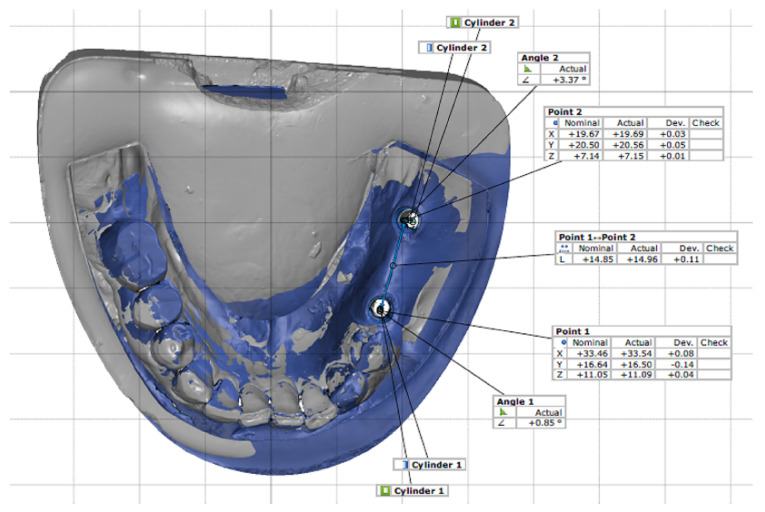
Impression measurements, inter-implant distance, linear, and rotational displacement by superimposition of nominal (standard) and actual (digital or conventional) STL files.

The STL files of three scans (reference model, conventional, and digital) were prepared. The two digital and conventional scans were superimposed on the standard model scan, and their accuracy was calculated by using the software’s best-fit algorithm for detailed 3D data assessment (GOM inspect v 7.5, GOM mbh, Braunschweig, Germany). The centre of the mesial implant platform was selected as point 1, and the centre of the distal implant platform was selected as point 2. The displacement and rotation of the implants were assessed in the x, y, and z axes. Thus, the files from the conventional and digital impressions were pre-aligned with the file from the standard model, and then the local best fit was applied. The discrepancies of the mesial implant were coded as x_1_, y_1_, and z_1_, and the changes in the distal implant were coded as x_2_, y_2_, and z_2_. The Δx^2^ + Δy^2^ + Δz^2^ = Δr^2^ formula was used to calculate and compare the linear displacement (∆r) [18,29]. To compare rotational changes, angle 1 referred to changes in the mesial implant, and angle 2 indicated the changes in the distal implant. In order to compare the inter-implant distance, the distance between point 1 and point 2 was measured and compared (Figure 1). 

### 2.8. Frameworks Passivity and Adaptation 

For the conventional screw-retained three-unit framework fabrication, two metal casting abutments (RAB45CN, Dentium Co., Seoul, Korea) underwent full-contour waxing, cut-back, and cast with cobalt–chromium (CoCr) alloy (Cara CoCr-SLM, Kulzer Co., Hanau, Germany). 

In the digital technique, a titanium (Ti) base abutment (Ti-007-NH, Arum Dentistry Co., Daejeon, Korea) was selected in 3Shape software (Dental system, 3Shape, København, Denmark), and a screw-retained monolithic zirconia framework (Katana translucent Zirconia, Kuraray, Bizen, Japan) was designed and machined using a 5-axis milling machine (Amann Girrbach, Koblach, Austria). The Ti-base was extraorally luted to the zirconia reconstruction using a temporary luting system (Temp-Bond, Kerr, Kloten, Switzerland). 

**Figure 2:**
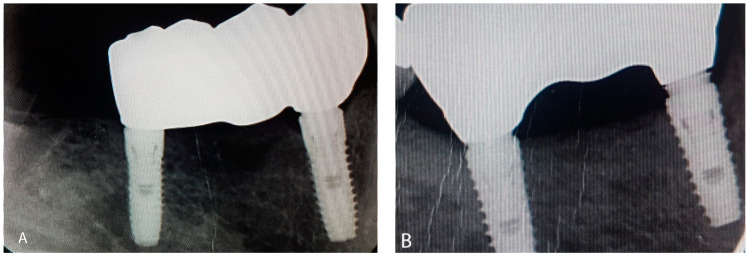
Framework try-in. (**A**) Acceptable framework; (**B**) unacceptable framework.

Both workflows consisted of a framework with an anatomical design, and their connectors had the same dimensions (i.e., height and width). The one-screw test (AKA, Sheffield test) and screw resistance test were used for assessing framework fitness and passivity [30]. In the one-screw test, one screw of the framework was torqued to 10 Ncm, and a parallel digital periapical radiograph was obtained. A custom-made acrylic bite jig standardized the radiographs for each patient [31,32]. All frameworks were qualitatively assessed (acceptable or unacceptable) by two evaluators (M.A., A.M.). Acceptable and unacceptable frameworks are shown in Figure 2a,b, respectively. A third evaluator acted as a tiebreaker in case of disagreement between evaluators’ assessments. 

**Figure 3:**
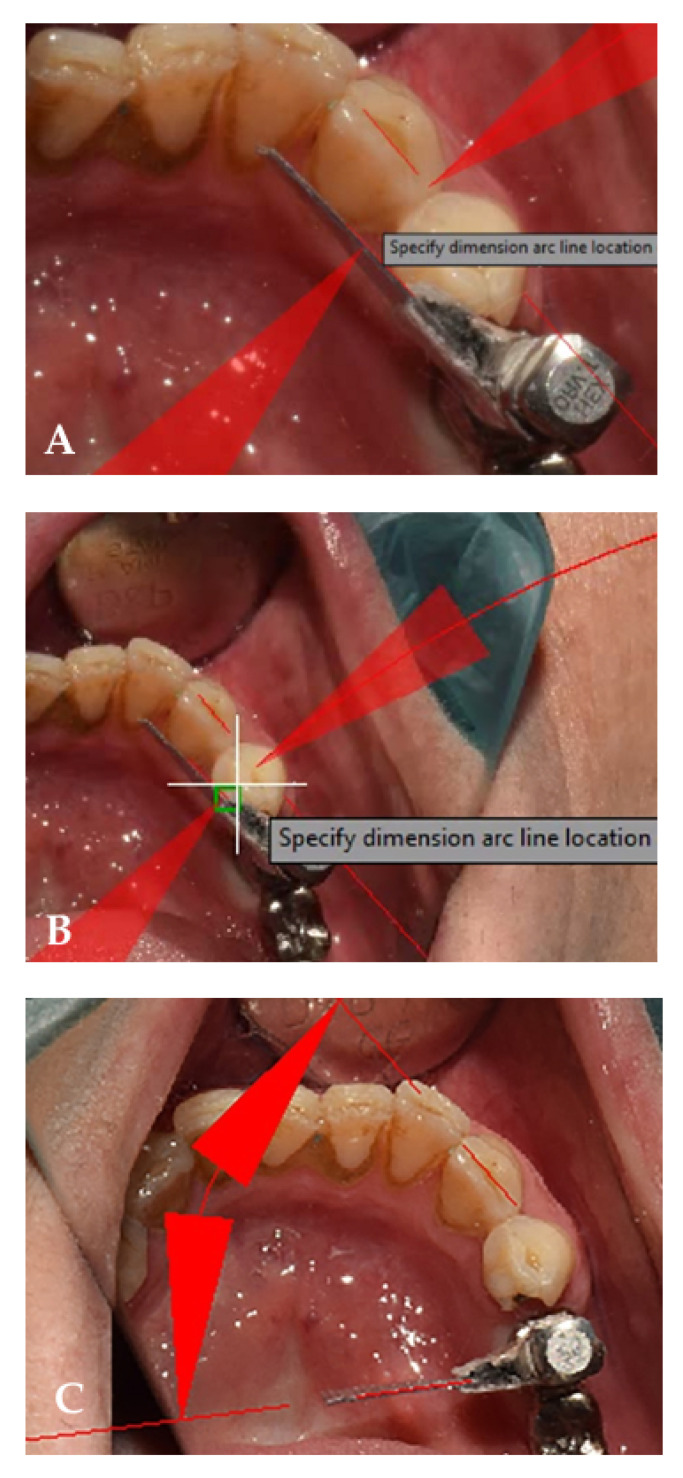
(**A**) A reference point on canine for calculation of the degree of the tape; (**B**) calculation of angle of movement of the tape before applying torque. This image displays 2 degrees of flag rotation; (**C**) calculation of angle of movement of the tape after applying torque. This image displays 56 degrees of flag rotation.

The flag technique was used for quantitative measurements and comparing the screw resistance test [30]. Consequently, adhesive tape (10 mm^2^) was placed on the head of the screwdriver, and the framework screw was tightened until initial resistance between the head of the screw and the framework was encountered. An intraoral photograph was taken from the assembly (Figure 3a). Next, the screw was torqued to 25 N/cm, and a second photograph was taken (Figure 3b). The two photographs were superimposed, and the angle of the tape between the two screwdriver positions was calculated (Figure 3c) and reported in degrees using computer-aided design (CAD) software (AutoCAD software; M.49.0.0 AutoCAD 2016, Autodesk, Inc., San Rafael, CA, USA). Additionally, the angle values between the digital and conventional techniques were compared.

### 2.9. Occlusal Assessment 

The conventionally-fabricated frameworks were veneered with leucite-reinforced feldspathic porcelain (Vintage PRO; Shofu INC, Tokyo, Japan), whereas the digital group consisted of full-contour zirconia reconstructions. The occlusion assessment included adjusting the proximal contacts to try in the ISFDPs, followed by adjusting the maxillomandibular contacts in centric occlusion. The duration of the occlusal adjustment process was recorded, and a jaw relation record was registered using an elastomeric material (Futar D, Kettenbach, Eschenburg, Germany). The elastomeric bite record was scanned with an industrial reference scanner (Atos Core 5 Mp 80 mm; Rev. 02; GOM GmbH, Braunschweig, Germany); 0 to 350 µm thickness of elastomeric bite record indicated optimal occlusal contacts [33]. Accordingly, the contact thickness of the two reconstruction workflows was compared. The thicknesses of 10 of the smallest contact points in the functional cusps of the ISFDPs were calculated and compared by the software for detailed 3D data assessment. The elastomeric bite records obtained from both workflows from each patient were superimposed for comparison purposes. Next, the functional area (i.e., the functional cusp slope, the cusp tip, and the tooth centre) was outlined and 10 of the smallest contact point thicknesses with ≥1 mm were measured and compared. 

### 2.10. Objective and Subjective Aesthetic Assessment 

The digital frameworks were extraorally luted to the Ti-base using an adhesive luting system (Multilink, Ivoclar Vivadent AG, Schaan, Liechtenstein). Both digital and conventional frameworks were stained and glazed. Each patient subjectively scored the appearance of their ISFDPs using visual analogue scale (VAS) [34], and two authors (MA, AMH) objectively assessed the aesthetics of the participants’ ISFDPs according to the FDI World Dental Federation aesthetic criteria [35]. A third evaluator contributed to the objective assessment when consensus was not reached. The FDI criteria consisted of the assessment of four aesthetic parameters for reconstructions, including shade colour match, translucency, lustre, and anatomical form. 

### 2.11. Delivery and Follow-Up

The ISFDP with superior parameters was delivered to each patient based on prosthesis passivity and adaptation followed by occlusion and aesthetic properties. The duration of treatment was calculated as the sum of four clinical and three laboratory sessions [36], and the average time (in minutes) was compared. Follow-up visits were carried out at 3 and 6 months. The patients were evaluated for any complications, including screw loosening, ceramic chipping, or delamination. 

### 2.12. Power Calculation and Sample Size 

The primary outcome of this study was the accuracy of digital and conventional impression techniques. Other comparisons were secondary outcomes. Therefore, based on the primary outcome, the sample size was calculated. The sample size calculation was in accordance with Amin’s study [37]. It was determined that 8 participants should be the minimum number of included patients in each group, assuming the mean and standard deviation of the accuracy of digital and conventional impression techniques to be 167.93 ± 50.37 µm and 46.41 ± 7.34 µm, respectively, to find a 50 µm difference between the two groups with alpha set at 0.05, and study power of 80%. Assuming a drop-out rate of 20%, 10 subjects were enrolled in each group. 

### 2.13. Statistical Analysis 

An independent statistician with expertise in dental medicine was blinded to the group allocation during the data analysis. The two workflows were compared with the Student *t*-test regarding linear displacement and inter-implant distance since the data distribution was deemed normal after the Shapiro–Wilk test (*p* > 0.05). The general estimating equation (GEE) model was applied to analyse the rotational displacement of implants statistically. Fisher’s exact test analysed framework adaptation, and the Mann–Whitney U test compared framework passivity. The GEE model was used for comparing occlusion between workflows. The Mann–Whitney test was applied for the comparison of aesthetic parameters. Depending on the normality of data distribution, the Mann–Whitney test or *t*-test was used to compare the reconstructions’ fabrication time. Alpha was set at 0.05 for the statistical analyses’ significance. The Statistical Package Statistical analyses were performed with the Statistical Package for the Social Sciences (IBM SPSS version 24; Chicago, IL, USA).

## 3. Results

All the enrolled participants completed the study. The sample consisted of 10 participants, 7 females (70%), with a mean ± SD age of 47.1 ± 11 years. No one had a history of tobacco consumption or bruxism. All participants received three-unit ISFDPs in the posterior mandibular region. A total of 20 prostheses were fabricated (2 prostheses for each patient; 10 prostheses in conventional workflow and 10 prostheses in digital workflow). 

### 3.1. Comparison of Digital and Conventional Impressions

Table 1 presents the implants’ inter-implant distance and linear and rotational displacement in the two impression techniques. The results revealed no significant difference in linear displacement (*p* = 0.506) or inter-implant distance (*p* = 0.858) between the two impression techniques. There was no significant difference in rotational displacement between the two impression techniques either (*p* = 0.759). 

### 3.2. Comparison of Framework Fabrication (Fitness and Passivity)

Table 1 also presents the measures of central dispersion for the passivity of anterior and posterior implants. These showed no significant difference in the passivity of the anterior (*p* = 0.280) or posterior (*p* = 0.739) implants between the digital and conventional techniques. 

The framework fitness was acceptable in all 10 cases in the conventional and 8 out of 10 in the digital workflow. There was no significant difference in the fitness of the two techniques (*p* = 0.747).

### 3.3. Comparison of Occlusion

The mean contact thickness was 0.60 ± 0.56 microns in the conventional and 0.49 ± 0.39 microns in the digital technique. There was no significant difference between the two groups (*p* = 0.487, standard error: 0.16). 

### 3.4. Comparison of Aesthetics

In the subjective assessment (by the patients), the mean VAS score was 8.4 ± 0.97 for the conventional and 8.6 ± 0.52 for the digital technique, with no significant difference (*p* = 0.684). In the objective assessment (by the clinicians), the lustre of most restorations fabricated by the two techniques was rated good, and no restoration had poor or unsatisfactory lustre. Regarding translucency, most restorations had satisfactory and good translucency. The colour of most conventionally-fabricated restorations was rated good, while most digitally-fabricated restorations were rated excellent. The anatomical form was rated good for most restorations in both techniques. There was no significant difference in any aesthetic parameter between the two groups (*p* > 0.05, Table 2). 

### 3.5. Comparison of Treatment Duration

Table 3 compares the clinical time, laboratory time, and total fabrication time of restorations in the two techniques. There was no significant difference in the mean clinical time between the two techniques (*p* = 0.444); however, the mean laboratory time was significantly shorter in the digital workflow (*p* < 0.001). The results revealed no significant difference in the occlusal adjustment time between the two techniques (*p* = 0.143). It showed that the mean total restoration fabrication time was significantly shorter in the digital workflow (*p* < 0.001).

## 4. Discussion 

This crossover-design clinical study identified and evaluated the impression accuracy, occlusion, aesthetics, duration of fabrication, framework passivity, and adaptation of the mandibular posterior three-unit ISFDP by the use of digital and conventional workflows. Each of the parameters were discussed in detail. The linear and angular displacements were obtained to compare the impression accuracy of the conventional and digital techniques. The null hypothesis was accepted in this respect since the two techniques were not significantly different. 

The linear and angular displacements were compared to compare the impression accuracy of the conventional and digital techniques. The null hypothesis was accepted in this aspect since the two techniques were not significantly different. Many previous studies reported the comparable accuracy of digital and conventional techniques [38,39,40,41], although most of them had an in vitro design [19,20]. Digital impression technique has been recommended for single-unit restorations [23,24], while disagreements exist regarding multiunit restorations [18,28,29]. Alsharbaty et al. [28] assessed the accuracy of closed-tray conventional, open-tray conventional, and digital techniques (by Trios scanner) by comparing with a reference model obtained by splinting impression copings. They measured the reference models with a coordinate measuring machine and reported that the accuracy of the closed-tray conventional technique was the highest, while the digital technique had a significantly lower accuracy. The difference between the present results and those of Alsharbaty et al. [28] is due to the different measurement methods of impression accuracy since they used a coordinate measuring machine. 

In contrast, the present study used an industrial scanner to measure conventional casts (STL files of the casts were compared in the present study). The present results were in line with those of Papaspyridakos et al. [19] who also used an industrial scanner with 6 µm accuracy (IScan D103i; Imetric). The accuracy of industrial scanners is another critical factor to consider. The present study used a non-contact triple scanner, which operates based on blue light technology, and has 2 µm accuracy. A desktop scanner or an IoS (that directly makes a digital impression) can be used to obtain digital data [42]. Desktop scanners reportedly have a comparable or higher accuracy than IoSs [43,44,45,46,47]. Another important factor is using different scan bodies or IoSs with different accuracy levels. Trios 3Shape IoS was used in the present study, and the results were in agreement with those of Amin et al. [37], who used Cerec Omnicam and 3M True Definition IoSs for comparison with the conventional technique. Different IoSs have different accuracy levels [48]. Vandeweghe et al. [49] used four different IoSs (Lava COS, 3M, CEREC Omnicam, Trios 3Shape) to make impressions from an edentulous mandible. They reported that 3M and Trios 3Shape had a higher accuracy than others. Gedrimiene et al. [29] reported the comparable accuracy of digital impressions made with the Trios 3 scanner and splinted open-tray conventional impressions. However, they used the conventional cast as the reference, while we fabricated a reliable, clinically acceptable reference model and compared both digital and conventional casts with the standard cast. 

Implant angulation is another fundamental factor affecting impression accuracy [41]. Generally, the accuracy of digital impressions is not influenced by implant angulation. Thus, the digital approach has eliminated concerns regarding introducing inaccuracies to the casts from impression material deformation or impression coping displacement [18]. However, Lin et al. [50]. reported that inter-implant angles up to 45 degrees did not affect the accuracy of conventional casts. In the present study, the inter-implant angle was less than 10 degrees. Moreover, a highly reliable “best-fit algorithm” was applied for superimposition [51]. 

The processes of cast fabrication and the type of restoration material are among other factors influencing the adaptation of definitive fixed reconstructions [33]. There is a great misfit risk for both cement-retained and screw-retained reconstructions. Although a marginal misfit of 10 to 150 µm is reportedly acceptable, no validated clinical threshold is available [52] since single-unit [41,53], three-unit (tooth-borne) [54,55], three-unit (implant-supported) [56], and full-arch [57,58] framework distortion mostly occurs in the conventional laboratory process phases, and the digital technology has a superior framework fit. Nonetheless, the present study found no significant difference in the passivity and adaption of digitally- and conventionally-fabricated three-unit frameworks. Single-unit frameworks fabricated by the conventional technique reportedly have higher accuracy than digitally fabricated types, which may be attributed to less technician involvement in the digital fabrication process [41,53]. Berejuk et al. [56]. used an optical comparator microscope to measure the microgap of a three-unit implant-supported framework and reported that all frameworks had a micro-gap < 70 µm; also, the amount of microgap in the digital groups was significantly lower than the conventional group. Abdel-Azim et al. [20] reported the same result for full-arch frameworks. They attributed this finding to the high technical sensitivity of the conventional fabrication of full-arch frameworks. In the present study, prosthetic adaptation was determined with periapical radiography. Clinical techniques (tactile sense, radiography, visual inspection) for measuring misfits may not detect gaps smaller than 50 µm, whereas gaps larger than 150 µm can be easily identified. Passivity is another important factor, which is imperative for successful long-term osseointegration [59]. Non-passive frameworks could lead to mechanical failure and peri-implant biological complications [60]. 

One of the fields that requires more clinical research is occlusion [30,56,59,61,62]. Assessment of the occlusion of digitally-fabricated restorations can reveal the better mounting accuracy of the digital technique [63,64] as bite registration is performed without human involvement in the digital technique [10]. In the present study, no significant difference between the conventional and digital techniques in the occlusal assessment (bite registration accuracy) indicated that the null hypothesis was accepted. However, some studies [61,65] reported that the digital technique failed to create uniform occlusal contacts, which may be due to the lower accuracy of the digital–physical cast compared with the conventional cast, attributed to the different accuracy of scanners [62]. In the present study, a digital–physical cast was not fabricated, and digital bite registration of the digital restoration was performed directly in the oral cavity. In this study, the optimal contacts were defined as homogenous and with lower mean thickness contacts.

Aesthetics’ assessment is an integral part of clinical studies [66]. Clinical aesthetic results should be evaluated both objectively by the clinician and subjectively by the patient [67,68,69]. Acceptable aesthetics of restorations depends on several factors, such as the anatomical form of the crown, translucency, lustre, and colour match of the restoration with the adjacent teeth [35]. In the present study, the digital and conventional techniques were not significantly different in terms of aesthetics for both patients and clinicians. Thus, the null hypothesis was accepted. 

Both patients and clinicians highly favour fewer appointments and shorter treatment duration [6,70,71,72]. In the present study, the clinical duration of the conventional and digital techniques had no significant difference, whereas the laboratory phase digital technique was significantly faster than the conventional technique. Thus, the null hypothesis was partially rejected. The fabrication time of fully digital monolithic lithium disilicate restorations with layered digital lithium silicate crowns (mixed digital and conventional workflow) reported that the fully digital workflow was faster due to less human manipulation and the non-use of time-consuming steps (e.g., porcelain powder application in the fully digital technique) [21]. The occlusal adjustment time was almost the same in the two techniques in the present study. In this study, both prostheses were made by a skilful dental technician. Human skill is more important for the conventional forming of porcelain powder on the occlusal surface than its digital design. Repeating this study with different levels of operator’s proficiency could have different results. 

In this study, one of the two prostheses was delivered to the patients, and one was kept as a backup. Therefore, it was not possible to compare their function in the long term or to assess the possible complications such as chipping and fracture. Another limitation was that the clinicians were not blinded when assessing the restorations, especially when assessing aesthetics. Increasing trends in using digital workflows make further clinical studies necessary. 

## 5. Conclusions

Most of the assessed outcomes of the fully digital workflow for the fabrication of mandibular posterior three-unit implant-supported fixed reconstructions were comparable to the conventional workflow. Thus, within the limitations of this clinical study, it is concluded that the fully digital workflow may be as reliable as the conventional workflow. More studies with a longer follow-up are required to confirm the favourable short-term outcomes observed with the digital workflow.

## Figures and Tables

**Table 1:** Description of inter-implant distance variation (µm), linear displacement (µm), rotational displacement (degree), and central dispersion for the passivity of mesial and distal implants of the two workflows.

Clinical Variables		Mean (SD) of Impression Techniques		*p*-Value
		Digital	Conventional	
**Impression**	*Inter-implant distance*	0.06 (0.15)	0.05 (0.11)	0.86
	*Linear displacement*	0.19 (0.09)	0.17 (0.06)	0.51
	*Rotational displacement*	2.19 (2.38)	2.4 (1.75)	0.76
**Passivity**	*Mesial implant*	74.5 (46.65)	93.1 (50.92)	0.28
	*Distal implant*	78.1 (34.47)	75.2 (32.06)	0.74

SD, standard deviation.

**Table 2:** FDI’s clinical criteria to assess aesthetic parameters (percentage).

Aesthetic Properties	Lustre		Colour Matching		Translucency		Anatomic Form	
	*Con*	*Dig*	*Con*	*Dig*	*Con*	*Dig*	*Con*	*Dig*
Clinically excellent/very good	10	10	40	50	10	0	0	10
Clinically good	50	80	50	20	40	70	70	70
Clinically sufficient/satisfactory	40	10	0	20	50	30	30	20
Clinically unsatisfactory	0	0	10	10	0	0	0	0
Clinically poor	0	0	0	0	0	0	0	0
*p*-Value	0.32		0.97		0.63		0.53	

Con, conventional; Dig, digital.

**Table 3:** Comparison of the clinical, laboratory, and total fabrication time (minute) of restorations in the two groups.

Procedure Time	Mean (SE)		*p*-Value
	*Conventional*	*Digital*	
**Clinical**	41.4 (2.02)	43.3 (1.35)	0.44
**Laboratory**	457.2 (3.61)	134.9 (1.56)	<0.01
**Total**	498.9 (4.50)	178.2 (2.60)	<0.01

SE: standard error.

## Abbreviations

ISFDPs: implant-supported fixed dental prostheses; IoS: intraoral scanner; PVS: polyvinylsiloxane; STL: stereolithography; CAD–CAM: computer-aided design–computer-aided manufacturing; 3D: three-dimensional; CONSORT: Consolidated Standards of Reporting Trials; VAS: visual analogue scale; GEE: general estimating equation.
